# Lactate Is a Major Promotor of Breast Cancer Cell Aggressiveness

**DOI:** 10.3390/cancers17111793

**Published:** 2025-05-27

**Authors:** Maitham A. Khajah, Sarah Khushaish, Yunus Luqmani

**Affiliations:** 1 Department of Pharmacology and Therapeutics, College of Pharmacy, Kuwait University, P.O. Box 24923, Safat 13110, Kuwait; sara.khochaich@ku.edu.kw; 2 Department of Pharmaceutical Chemistry, College of Pharmacy, Kuwait University, P.O. Box 24923, Safat 13110, Kuwait; yluqmani@yahoo.com

**Keywords:** breast cancer, EMT, lactate, endocrine resistance, motility, breast cancer, endocrine resistance, lactate, LDH, proteomic profiling

## Abstract

**Background**: Lactate dehydrogenase (LDH) activity, producing high levels of lactate from pyruvate in cancer cells, is often associated with poor patient prognosis. We previously showed enhanced LDH/lactate levels in estrogen receptor (ER) compared to ER + breast cancer cells; lactate or pyruvate supplementation to ER + cells significantly enhanced their motile ability, while LDHB gene knockout (KO) or treatment with LDH inhibitors reduced the motility of the highly aggressive ER breast cancer cells. **Aims**: To investigate the molecular mechanisms by which lactate, LDHB KO, or treatment with LDH inhibitors can modulate the motile capabilities of breast cancer cell lines. **Methods**: KO experiments were performed using siRNA, and global expression was determined by proteomic profiling with Proteome Profiler Human XL Oncology arrays, Western blot, and immunofluorescence. **Results**: Lactate supplementation to ER + breast cancer cells enhanced expression of vimentin, N-cadherin, and snail, while reducing the expression of JAM-A, E-cadherin, and nectin-4. This expression profile was reversed with LDHB KO in ER cells. LDHB KO, or treatment with LDH inhibitors in ER cells, also reduced the expression of IL-6, IL-8, and MMP-2. The expressions of other markers such as PECAM-1, CCL20, and ENPP-2 were differentially modulated with LDH B KO in de novo ER cells (MDA-MB-231) vs. those that had ER knockout (pII). **Conclusions**: Our data show a novel role for lactate in modulating the EMT status in breast cancer cells and highlight the important role of lactate in breast cancer motility in part through modulating EMT status and the expression profile of cytokines, adhesion molecules, MMP-2, and nectin-4.

## Simple Summary

Breast cancer cells produce high amounts of lactate in the tumor microenvironment, and we believe that this end-product plays an important role in cancer cell invasion. Herein, we showed experimental evidence that lactate supplementation to estrogen receptor-positive breast cancer cells (which have low motile and invasion profile) enhanced their aggressiveness through increased expression of mesenchymal proteins. On the other hand, decreased lactate production in endocrine-resistant breast cancer cells (which have high motile and invasive profile) reduced their aggressiveness through reduced expression of mesenchymal protein and enhanced expression of epithelial proteins. These data allow better understanding for the role of lactate in breast cancer pathogenesis. It may be a possible future therapeutic option in cancer treatment.

## 1. Introduction

Over recent decades, the understanding of cancer has evolved from a purely genetic perspective, focusing on mutations and oncogenes, to re-positioning it as a disease driven by altered metabolic processes. Although not without controversy, this viewpoint envisions the metabolic environment of a tumor as a significant influence on cancer progression, including its potential for metastasis. Central to this metabolic framework is the prominence of lactate, produced from pyruvate by lactate dehydrogenase (LDH), a ubiquitously expressed cytosolic enzyme. LDH has three minor and two predominant translated isoenzyme forms which exhibit a varied tissue expression profile. Principally, LDH-A is expressed in skeletal muscle [1], while LDH-B is mainly expressed in the heart and brain [1]. In normal cells, lactate is produced as a temporary metabolite under transient anaerobic conditions; when oxygen levels are restored, it is converted back to pyruvate for entry into the TCA cycle to produce ATP by oxidative phosphorylation. Cancer cells apparently forego this conversion even in the presence of sufficient oxygen, deriving their ATP principally from glycolysis, an energetically inefficient (and still perplexing) phenomenon first described by Warburg in the 1920s [2], hence the increased (and therefore presumably reliance upon) high glucose consumption by tumors.

LDH expression is significantly enhanced in several tumors including breast, pancreatic, colorectal, and squamous cell head, and neck cancer [3,4,5,6,7,8]. LDH-A upregulation is often associated with poor prognosis and increased metastatic potential [9]. LDH facilitates not only increased lactate production but also the downstream metabolic adaptations necessary for sustaining high proliferation rates, such as accelerated nucleotide biosynthesis through the pentose phosphate shunt and fatty acid synthesis [10,11]. Moreover, lactate extruded by tumor cells can be utilized by adjacent stromal cells, promoting their survival, and contributing to a metabolic symbiosis that could facilitate tumor progression.

As the major end product of glucose metabolism, lactate extrusion, to prevent cellular acidosis, leads ostensibly to an acidic extracellular tumor microenvironment due to the co-transport of H^+^ [12]. Many recent reports have highlighted the role of lactate as an energy fuel, particularly for cancer cells within the oxygen-deprived center of a large tumor. Higher tumor grade has been associated with increased lactate concentrations in breast cancer patients [13,14]. In lung cancer, lactate uptake enhances the oxidative pentose phosphate pathway leading to enhanced cancer cell metastasis [15,16,17]. Lactate was also reported to confer cancer cell resistance various drugs [18] and to glucose deprivation-induced death, in part through inhibiting apoptosis and enhancing autophagy [19]. In breast cancer, the induction of tamoxifen resistance in MCF7 cells enhanced LDH activity and lactate production [20]. Furthermore, lactate contributes to the modulation of the immune response towards cancer cells, promoting immunosuppression [21].

In view of these many observations, it is evident that the role of lactate in cancer progression has been greatly under-appreciated. Most attention has been focused on extracellular acidity, produced as a result of proton co-transport with lactate extrusion, as being the driving force for tumor progression. Our previous in vitro studies [22,23] suggest this may be an erroneous distraction. We provided evidence to show that MCF7 breast cancer cells which have undergone induced epithelial to mesenchymal transition (EMT; now considered part of the mechanism involved in tumor metastasis [24,25]), as a consequence of shRNA induced estrogen receptor (ER) down-regulation, display increased propensity for motility and invasion. These properties are promoted, in addition to other described factors, by alkalization of the extracellular environment leading to plasma membrane blebbing, increased matrix metalloproteinase production and increased extracellular lactate [26,27,28].

We demonstrated enhanced total LDH activity and lactate production in the transformed ER compared to the parental ER + MCF7 breast cancer cell lines or normal breast epithelial cells [23]. The LDH-A isoenzyme is expressed in both ER + and ER- cells, while LDH-B is specifically expressed only in ER cells. Also, lactate or pyruvate supplementation to the slowly motile MCF7 cells significantly enhanced their motile (but not proliferative) ability, concurrently with enhanced EKR1/2 phosphorylation [23]. Also, we showed that siRNA-induced silencing of either LDH-A or LDH-B genes in ER- or ER + breast cancer cells, or the use of pharmacological LDH inhibitors to reduce LDH activity, resulted in reduced extracellular lactate and cell motility. These data support the notion that lactate plays an important role in transforming poorly motile breast cancer cells into a more aggressive phenotype [22,23]. Reports from other authors also propose that lactate could function as a signaling molecule that can influence various cellular processes, including proliferation, migration, and immune evasion [29,30]. Consequently, the accumulation of lactate within the extracellular microenvironment may facilitate a pro-tumorigenic condition that supports not only local tumor growth but also metastatic dissemination. Inhibiting LDH has demonstrated anti-tumor effects in preclinical models [1], but the systemic effects of such interventions could also lead to disruption of essential metabolic processes in normal tissues.

In the study reported here, we investigated the molecular mechanisms by which lactate could modulate the motile capabilities of different breast cancer cell lines by examining the proteomic expression profile in cells that have been treated with lactate, as well as in cells in which the LDH B gene is silenced (using siRNA) or treated with LDH inhibitors. Our data suggested that lactate supplementation in ER + breast cancer cells enhanced their motile abilities in part through increased expression of various mesenchymal markers with reduction in some epithelial markers. This expression profile was reversed in ER- breast cancer cells upon silencing of the LDHB gene. In addition, LDHB KO or treatment with LDH inhibitors in ER- breast cancer cells reduced their motile ability in part through reducing the expression of IL-6, IL-8, and MMP-2. These data highlight the novel role for lactate in modulating the EMT status and subsequent modulation in cell aggressiveness (such as cell motility), which was mediated in part through modulating the expression of various cytokines, adhesion molecules, MMP-2, and nectin-4.

## 2. Materials and Methods

### 2.1. Cell Lines

The endocrine responsive ER + MCF7 and the de novo endocrine-resistant ER- MDA-MB-231 breast cancer cells were obtained from the American Type Culture Collection (ATCC, Manassas, VA, USA). The acquired endocrine-resistant ER- breast cancer cell line pII was established in our laboratory by transfecting the parental MCF-7 with an shRNA plasmid targeting the ER sequence as previously described [31]. The YS1.2 (ER +) cell line is a similarly transfected MCF7 derivative that failed to downregulate ER, maintaining its ER expression; this was used as a transfected control for the pII cells [32]. For routine culture, all cell lines were maintained as monolayers in advanced Dulbecco’s minimum essential medium (DMEM) containing phenol red and supplemented with 5% fetal bovine serum (FBS), 600 µg/mL L-glutamine, 100 U/mL penicillin, 100 µg/mL streptomycin, and 6 mL/500 mL 100 × non-essential amino acids (all from Invitrogen, Carlsbad, CA, USA), and they were grown at 37 °C in an incubator gassed with an atmosphere of 5% CO_2_ and maintained at 95% humidity. Cell cultures were periodically treated with mycoplasma removal agent from Biorad (Hercules, CA, USA) and tested with detection kits from Invivogen (Carlsbad, CA, USA) and DAPI nuclear staining to ensure they remained free of mycoplasma.

### 2.2. siRNA-Mediated Knockdown of LDHB mRNA

Cells were seeded into 12-well plates and cultured for 24 h to reach 60–80% confluency before transfection using Lipofeactamine RNAiMAX reagent. The procedure was performed according to the manufacturer’s protocol. In brief, solution A (100 μL of optiMEM + 6 μL reagent) and solution B (100 μL of optiMEM + 20 pmol of siRNA) were prepared, mixed gently, and incubated for 15 min at room temperature. For each well, this complex was added dropwise to the wells containing 1 mL DMEM media and placed in a 37 °C, 5% CO_2_ incubator for 48 and 72 h. Knockdown of LDHB mRNA was confirmed by Western blotting [33]. LDHB Human siRNA Oligo Duplex was purchased from Locus ID3945 (Cat # SR320835).

### 2.3. Western Blotting

Cells were cultured in 6-well plates to 80–90% confluence. The medium was aspirated off and cell monolayers were harvested by scraping and re-suspension into 300 μL of lysis buffer containing 50 mM HEPES, 50 mM NaCl, 5 mM EDTA 1% Triton X, 100 µg/mL phenylmethylsulfonyl fluoride (PMSF), 10 µg/mL aprotinin, and 10 µg/mL leupeptin. Lysates were stored at −80 °C until use. Protein concentration was determined by the Bradford assay using BSA as standard; 8 μg protein lysate was mixed with an equal volume of 2 × SDS and heated at 90 °C for 10 min. Samples were loaded onto a 10% SDS-polyacrylamide gel and electrophoresed at 150 V for 1 h. Proteins were then transferred onto nitrocellulose membranes and blocked with 2% BSA for 1 h before being incubated overnight at 4 °C with different primary antibodies (all at 1:1000 dilution in 5% BSA): β-Actin (13E5) Rabbit mAb (Cat # 4970, from Cell Signaling, Danvers, MA, USA), LDHA (C4B5) Rabbit mAb (Cat #3582, from Cell Signaling), LDHB (Cat # PB10076, from Picoband™), and E-Cadherin (24E10) Rabbit mAb (Cat #3195, from Cell Signaling). Membranes were then washed and incubated with Anti-rabbit IgG, HRP-linked Antibody (Cat # 7074, from Cell Signaling) at 1:1000 dilution in 5% BSA for 1 h; after development with Super Signal ECL (Thermo Scientific, Rockford, IL, USA), bands were visualized with a Cell bio-imager (ChemiDoc MP System from BioRad, Hercules, CA, USA).

### 2.4. Immunofluorescence

Cells were seeded at approximately 5000/well in an 8-well chambered slide containing 300 µL/well DMEM medium and cultured to 80% confluence. The medium was then removed, and cells were immediately fixed by addition of 3.7% formaldehyde (200 µL/well) with gentle agitation for 10 min. Non-specific binding sites were blocked by addition of 1% BSA in PBS (100 µL), and cells were incubated at room temperature for 1 h. The BSA was then aspirated and 200 µL/well of one of the primary antibodies (E-Cadherin (24E10) Rabbit mAb (Cat #3195, from Cell Signaling), JAM-A (1H2A9), Cat # sc-53624, from Santa Cruz, Dallas, TX, USA), N-cadherin (D4R1H; Cat #13116, from Cell Signaling), Vimentin (D21H3; Cat #5741, from Cell Signaling), and snail (C15D3; Cat # 3879, from Cell Signaling) was also added (at 1:100 dilution). The obtained solution was left to incubate overnight at 4 °C. Cells were then washed with PBS and the secondary antibody (Anti-rabbit IgG Alexa Fluor^®^ 555 Conjugate, Cell Signaling, Danvers, MA, USA) (1:500 dilution) was added. Incubation continued for 2 h at room temperature in the dark. Following washing steps with PBS, 200 µL/well of diluted phalloidin (Thermo-fisher, Waltham, MA, USA) (6 µL in 1 mL PBS) was added and incubated for 10 min at room temperature in the dark. After further washing with PBS three times, the chambers and borders of the 8-well chambered slides were removed and a drop of diamidine phenylindole dihydrochloride (DAPI) was added onto the slide prior to mounting. Staining was visualized and photographed using an LSM 510 Meta confocal microscope (Carl Zeiss, Oberkochen, Germany) at an excitation wavelength of 450 nm.

### 2.5. Protein Profiling

About 10^6^ cells/well were cultured in a 6-well plate with the various treatment conditions.

Following washing with ice-cold PBS, cells were lysed using Pierce ^TM^ lysis buffer supplemented with protease inhibitors (1 µg/mL leupeptin, 1 µg/mL aprotinin, and 10 µg/mL phenylmethylsulfonyl fluoride [PMSF]) and harvested using a rubber cell scraper. Lysates were centrifuged at 14,000× *g* for 10 min, and the supernatant stored at −80 °C for subsequent analysis. Total protein concentration was determined by the standard Bradford assay.

The expression profile of 84 proteins involved in cancer pathogenesis was determined using the Proteome Profiler Human XL Oncology Array (Cat # ARY026, R&D Systems, Minneapolis, MN, USA) according to the manufacturer’s procedure. First, nitrocellulose membranes were blocked with 2 mL array buffer 6 in a 4-Well Multi-dish for 1 h. Cell lysates (200 μg protein) were diluted with 0.5 mL of array buffer #4 and volume was adjusted to 1.5 mL with assay buffer #6. Diluted samples were added to the blocked membranes after the removal of buffer and incubated overnight at 4 °C. Membranes were then washed three times for 10 min each with 20 mL 1× washing buffer and then incubated with 2 mL of the diluted detection antibody mix for 0.5 h. Membranes were then washed again as described above and placed into a 4-Well Multi-dish containing 2 mL of diluted streptavidin-HRP for 0.5 h at room temperature. Membranes were then washed and incubated in 1 mL of the chemi-reagent mix for 1 min at room temperature. Using the supplied transparency overlay template, positive signals were identified on the membrane referring to the given appendix in the kit protocol manual. Spot intensity was quantified using the GS 800 calibrated densitometer (Quantity One software version 4.6.9, Bio-rad^®^) and the average of duplicate spots on each membrane were normalized with the average control spots provided for each membrane.

### 2.6. Statistical Analysis

Means of experimental groups were compared with controls using Student’s *t*-test or one-way ANOVA followed by *Bonferroni* post hoc test. Statistical significance was assumed at *p* values <0.05 using GraphPad Instat software. GraphPad Prism 6 was used to plot graphs.

## 3. Results

### 3.1. Effect of Lactate Treatment in ER + or LDHB KO in ER- Breast Cancer Cells on the Expression of Various Effector Targets

**Figure 1:**
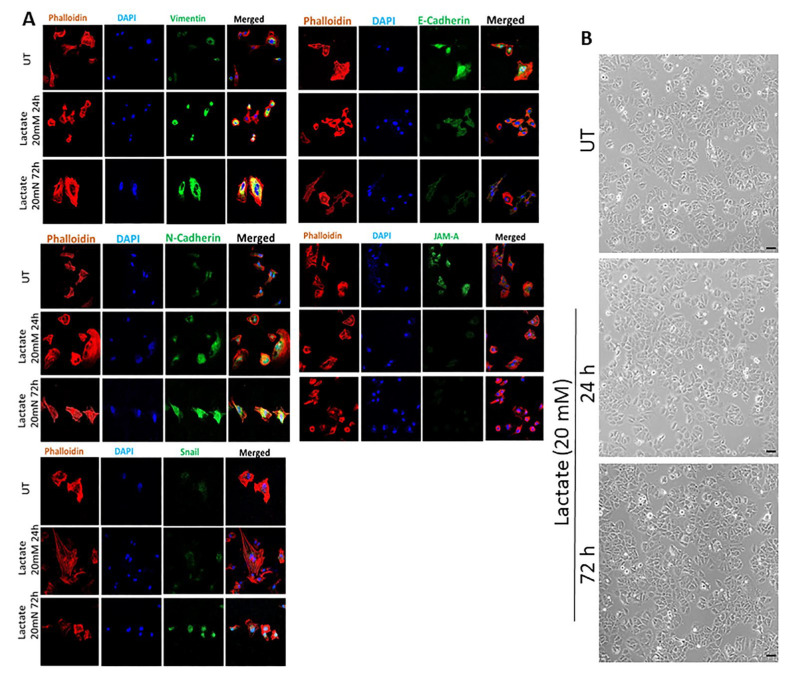
Effect of lactate treatment on the expression profile of EMT markers and JAM-A in ER + breast cancer cells. Panel (**A**) shows immunofluorescence analysis of YS1.2 cells, either untreated (UT) or in response to lactate treatment (20 mM, 24–72 h). Cells were stained with phalloidin (red for actin cytoskeleton), DAPI (blue for the nucleus), or various targets (green) (63× magnification). Panel (**B**) shows the effect of lactate treatment on cell morphology (20× magnification) up to 72 h. Scale bars represent 40 µm (n = 3 per group).

Exposure of YS1.2 ER + cells to lactate (20 mM, for 24–72 h) resulted in enhanced expression of various mesenchymal markers such as vimentin, N-cadherin, and snail. It also resulted in reduced expression of the epithelial marker E-cadherin and junctional adhesion molecule-A (JAM-A) (Figure 1A). The lactate-induced changes in the expression of the above-mentioned markers were not associated with morphological changes (Figure 1B).

**Figure 2:**
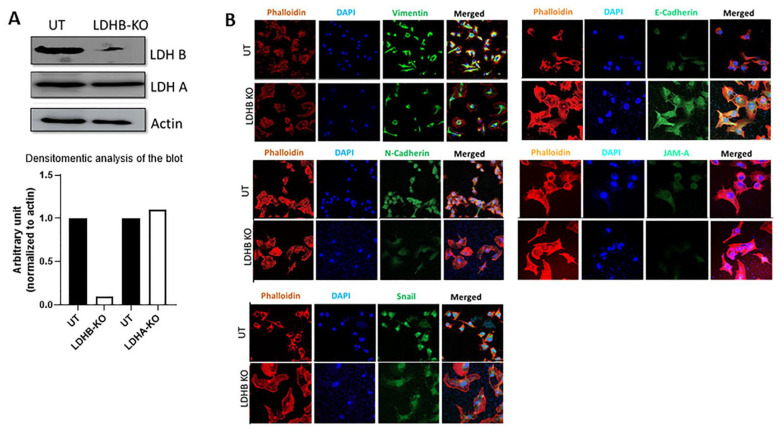
Effect of lactate treatment on the expression profile of EMT markers and JAM-A in ER- breast cancer cells. Panel (**A**) shows Western blot analysis for LDHA and LDHB in pII cells, either UT or in response to LDHB KO. Panel (**B**) shows immunofluorescence analysis of pII cells, either untreated (UT) or in response to LDHB KO. Cells were stained with phalloidin (red for actin cytoskeleton), DAPI (blue for the nucleus), or various targets (green) (63× magnification) (n = 3 per group). Original western blots are presented in File S1.

Conversely, LDHB KO in the ER- pII cell line (confirmed by Western blotting as shown in Figure 2A), which did not affect LDHA expression, resulted in reduced expression of vimentin, N-cadherin, snail, and JAM-A, and increased expression of E-cadherin (Figure 2B).

**Figure 3:**
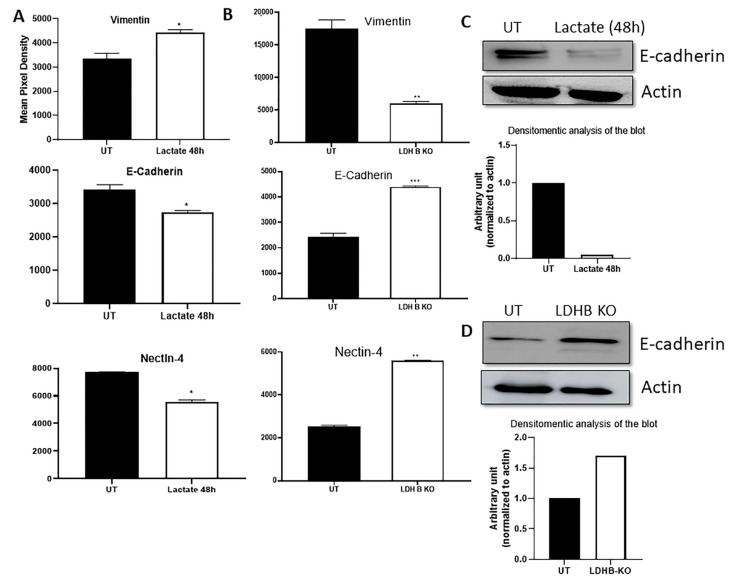
Effect of lactate treatment in YS 1.2 or LDHB KO in pII cells on the expression of vimentin, E-cadherin, and nectin-4. Densitometric analysis of protein profiling for various targets in YS1.2 (panel (**A**)) and pII cells (panel (**B**)), either UT (solid bars) or in response to lactate treatment (20 mM, 48 h) or LDHB KO (open bars). Asterisks denote the significant differences compared to UT with * *p* ˂ 0.05, ** *p* ˂ 0.001, *** *p* ˂ 0.0001 (n = 4 per group). Western blot analysis for E-cadherin is shown for YS1.2 cells, either UT or in response to lactate treatment (20 mM, 48 h, panel (**C**)), and for pII cells, either UT or in response to LDHB KO (panel (**D**)) (n = 3 per group). Original western blots are presented in File S1.

This inverted expression pattern between lactate treatment of YS1.2 and LDHB KO in pII cells for vimentin and E-cadherin was also confirmed by proteomic profiling (Figure 3A,B). We also observed decreased nectin-4 expression in YS1.2 cells with lactate treatment and increased expression in LDHB KO pII cells (Figure 3A,B). Western blot analysis was used to further confirm the modulated expression profile of E-cadherin in YS 1.2 in response to lactate treatment (Figure 3C) and in pII in response to LDHB KO (Figure 3D), as it is considered one of the most important molecules involved in the EMT process. The expression profile of several other targets was also modulated but not consistently across all cell lines examined (Supplementary Figures S1–S3).

### 3.2. Effect of LDHB KO or Treatment with LDH Inhibitors in pII and MDA-MB-231 Cells

**Figure 4:**
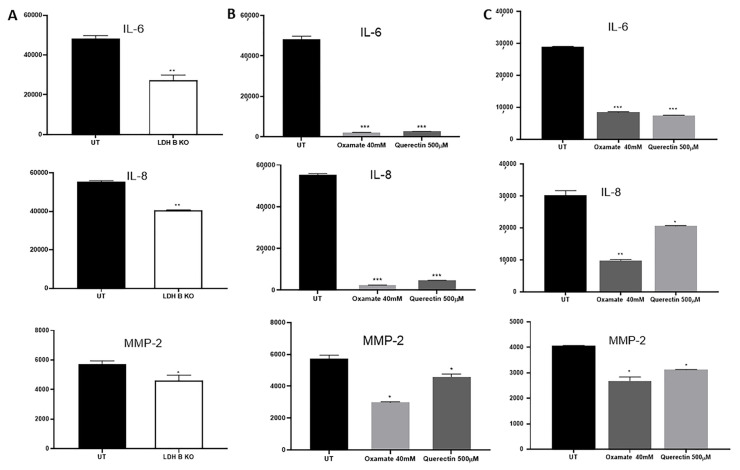
Effect of LDHB KO or LDH inhibition on the expression of IL-6, IL-8, and MMP-2 in ER- breast cancer cells. Densitometric analysis of protein profiling for various targets in pII (panels (**A,B**)) and MDA-MB-231 cells (panel (**C**)), either UT (solid bars), in response to LDHB KO (open bars), or LDH inhibition (gray bars). Asterisks denote the significant differences compared to UT with * *p* ˂ 0.05, ** *p* ˂ 0.001, *** *p* ˂ 0.0001 (n = 4 per group).

Using proteomic profiling, the expression profile of cytokines and proteinases involved in cell motility and invasion such as IL-6, IL-8, and MMP-2 were found to be significantly reduced in pII cells in response to LDHB KO (Figure 4A), and in pII (Figure 4B) and MDA-MB-231 (Figure 4C) cells in response to treatment with the LDH inhibitors quercetin and oxamate.

**Figure 5:**
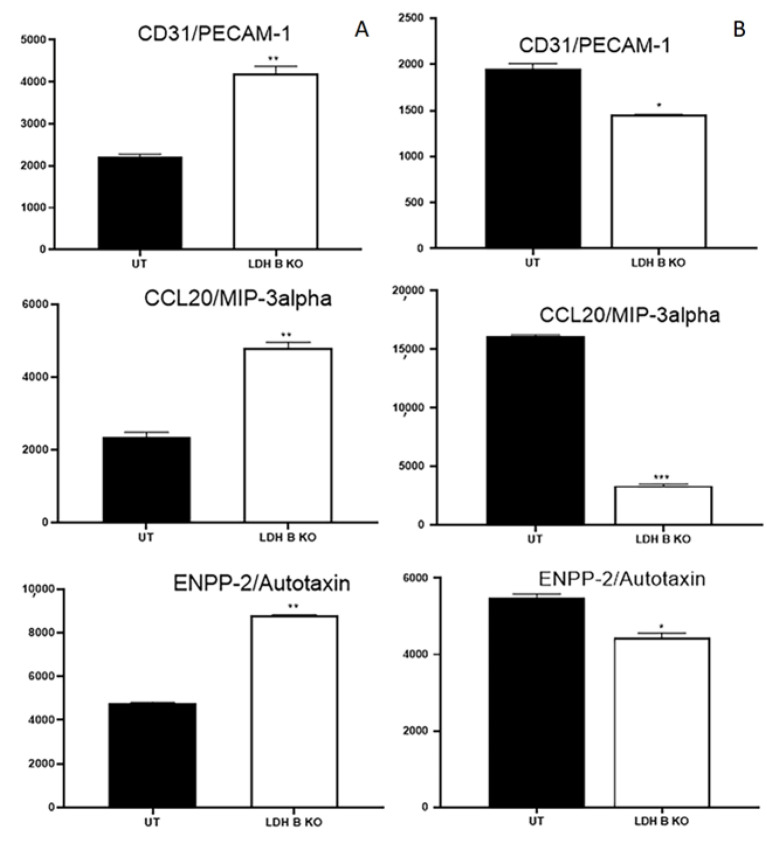
Effect of LDHB KO on the expression of CD31, CCL20, and ENPP-2 in ER- breast cancer cells. Densitometric analysis of protein profiling for various targets in pII (panel (**A**)) and MDA-MB-231cells (panel (**B**)), either UT (solid bars) or in response to LDHB KO (open bars). Asterisks denote the significant differences compared to UT with * *p* ˂ 0.05, ** *p* ˂ 0.001, *** *p* ˂ 0.0001 (n = 4 per group).

The expression profile of other markers was also differentially modulated between pII and MDA-MB-231 in response to LDHB KO; expression of CD31, CCL20, and ENPP-2 was increased in pII and decreased in MDA-MB-231 cells (Figure 5).

## 4. Discussion

We previously presented evidence that extracellular lactate plays an important role in enhancing breast cancer cell motility and invasion [22,23]. In this study, we show that this effect of lactate is in part through modulating the expression of various EMT markers and the expression profile of other important molecules involved in cell motility and invasion such as IL-6, IL-8, MMP-2, and nectin-4. Furthermore, there are some differences in the expression profile of other markers such as PECAM-1, CCL20, and ENPP-2 in response to LDHB KO between de novo and acquired forms of endocrine resistance.

The extracellular microenvironment has received increasing attention in recent years, with recognition of its pivotal influence on tumor dissemination. However, one of its major constituents that has been largely neglected is lactate. Though it is considered a waste metabolic product in hypoxia, we believe that lactate could be a major factor in promoting metastasis with significantly high concentrations found around the tumor (up to 40 mM) compared to physiological concentrations in blood and tissues (1.5–3 mM) [34,35]. Reports published in the 1980s by George Brooks demonstrated that lactate is the preferred fuel for most (including cancer) cells as well as an important signaling molecule [36,37,38]. Lactate was shown to elicit histone acetylation, acting as an epigenetic modulator, and increasing gene transcription from exposed chromatin in both human and mouse cells [39]. Also, lactate was reported to modulate cellular metabolism through histone lactylation-mediated genes in non-small cell lung cancer [40] and in cancer-associated fibroblasts from pancreatic ductal adenocarcinoma [41]. Moreover, lactate was shown to regulate cell cycle events by remodeling the anaphase promoting complex [42]. Lactate was shown to increase cell migration and invasion in part through inducing transforming growth factor-β2 (TGFβ2) expression [43]. High concentrations of lactate surrounding the tumor mass are reported to correlate with metastasis [44,45] in various forms of cancer including cervical [46], head and neck [47], gastric [48], and colorectal adenocarcinoma [49].

There is now substantial evidence that EMT, a complex multifactorial physiological process involved in wound healing and embryogenesis, also plays a vital role in cancer metastasis. Many proteins are involved in this process including E-cadherin, mucin, N-cadherin, vimentin, Snail, and ZEB1/2 [25,50,51]. Lactate was shown to promote EMT in gastric cancer cells by upregulating ZEB2 [52]. Furthermore, lactate treatment, at similar concentrations to those used in the current study, induced EMT, cell migration, and invasion in renal carcinoma cells by downregulating SIRT1 expression [53]. In human lung adenocarcinoma cells, lactate treatment (15–20 mM) enhanced tumor invasion through decreased E-cadherin expression [54]. Also, LDHA knockdown in lung cancer cells resulted in MET induction through decreased E-cadherin and increased vimentin, N-cadherin, Snail, and ZEB1 expression [55]. Using MCF-7 and MDA-MB-231 breast cancer cells, San-Millan et al. [56] showed that lactate levels were higher in ER- compared to ER + breast cancer cells, which confirms our previous findings [23]. Our data show that lactate treatment (20 mM for 24–72 h) in YS1.2 cells enhanced the protein expression profile of various mesenchymal markers such as vimentin, N-cadherin, and snail, and reduced the expression of the epithelial marker E-cadherin and JAM-A. The expression of the above-mentioned markers was reversed in pII cells with LDHB KO (Figure 1, Figure 2 and Figure 3). These data suggest that lactate enhanced cell motility in part through modulating the EMT status in breast cancer cells.

The adhesion molecule nectin-4 was shown to play a role in breast cancer pathogenesis, and its over-expression has been observed in various forms of cancers including breast, lung, colorectal, pancreatic, and ovarian cancers [57]. High expression of nectin-4 was reported to be associated with poor prognosis in part through enhancing cell proliferation, angiogenesis, EMT induction, and metastasis [57]. In triple-negative breast cancer cells, treatment with N41mab-vcMMAE comprising a human anti-nectin-4 monoclonal antibody conjugated to monomethyl auristatin-E (MMAE) resulted in inhibition of cell proliferation in vitro and reduced metastasis in vivo [58]. Also, a role for nectin-4 has been demonstrated in promoting tumor-induced lymphangiogenesis and lymphatic metastasis in part through modulating the CXCR4/CXCL12-LYVE-1-axis [59]. Zeindler et al., however, reported conflicting evidence suggesting that nectin-4 overexpression is associated with better survival in triple-negative breast cancer [60]. Herein, we showed that lactate treatment in ER + ve breast cancer cells reduced nectin-4 expression and LDHB KO in ER- cells enhanced its expression (Figure 3).

**Figure 6:**
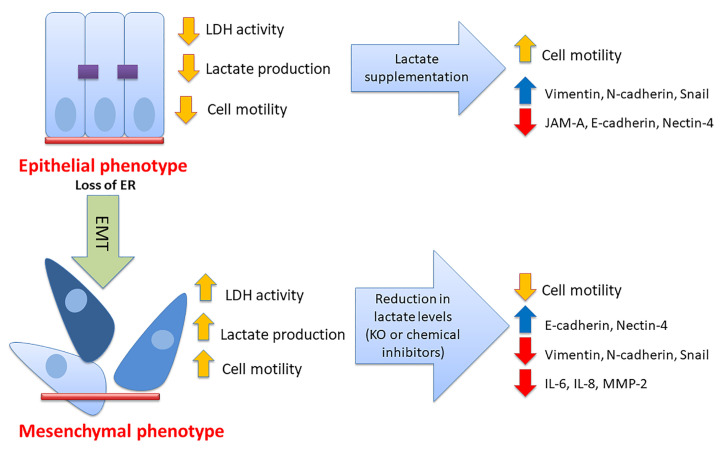
Model of the generated data. Lactate is considered an important played in breast cancer cell aggressiveness in part through modulating the EMT status and the expression of various adhesion molecules, proteinases, and cytokines.

Enhanced serum levels of IL-6 and IL-8 in breast cancer patients was associated with advanced clinical disease stage, lymph node metastasis, and poor prognosis [61,62]. Interestingly, IL-6 and IL-8 treatment induced EMT in MCF7 cells and was required to maintain the aggressive behavior of MDA-MB-231 cells [63]. Treatment with the humanized anti-IL-6 receptor tocilizumab (Actemra) (which is also a potent inhibitor of IL-8) in TNBC cells resulted in reduced cell aggressiveness in part through modulating the expression of vascular endothelial growth factor-A and hypoxia-inducible factor-1 both in vitro and in vivo [64]. Herein, we demonstrated reduced expression of IL-6 and IL-8 in response to LDHB KO or LDH inhibition in ER- breast cancer cells (Figure 4). The zinc matrix metalloproteinases (including MMP-2) are involved in the propagation of various types of cancers, including the breast [65]. MMP-2 plays a vital role in angiogenesis and metastasis. We also observed reduced expression of MMP-2 in response to LDHB KO or LDH inhibition in ER- breast cancer cells (Figure 4). We believe that the effect of lactate in promoting cell motility and invasion is not restricted to the producer cell (cancer cell, as partially described in the current study) but is more involved in modulating the cancer microenvironment (including extracellular matrix and immune cells), which requires further studies. Future perspectives for this work include the role of lactate in modulating the status of the extracellular matrix proteins and how this affects breast cancer cell motile and invasive behavior and resistance to endocrine therapy. The role of lactate in modulating other components of the microenvironment also warrants further investigation. A model describing the published data in this report is shown in Figure 6.

## 5. Conclusions

In conclusion, several reports by our group and others demonstrate that lactate plays an important role in breast cancer motility in part through modulating EMT status, and the expression profile of cytokines, adhesion molecules, MMP-2, and nectin-4.

## Data Availability

All data generated or analyzed during this study are included in this published article [and its Supplementary Materials].

## Supplementary Materials

The following supporting information can be downloaded at: https://www.mdpi.com/article/10.3390/cancers17111793/s1, Figure S1: Effect of lactate treatment on the expression of other markers in ER + breast cancer cells. Densitometric analysis of protein profiling for various targets in YS1.2 cells, either UT (solid bars) or in response to lactate treatment (open bars). Asterisks denote the significant differences compared to UT with * *p* ˂ 0.05, ** *p* ˂ 0.001 (n = 4 per group); Figure S2: Effect of LDHB KO on the expression of other markers in pII cells. Densitometric analysis of protein profiling for various targets in pII cells, either UT (solid bars) or in response to LDHB KO (open bars). Asterisks denote the significant differences compared to UT with * *p* ˂ 0.05, ** *p* ˂ 0.001 (n = 4 per group); Figure S3: Effect of LDHB KO on the expression of other markers in MDA-MB-231 cells. Densitometric analysis of protein profiling for various targets in MDA-MB-231 cells, either UT (solid bars) or in response to LDHB KO (open bars). Asterisks denote the significant differences compared to UT with * *p* ˂ 0.05, ** *p* ˂ 0.001, *** *p* ˂ 0.0001 (n = 4 per group).
