# Supplementary material for: Lactate Is a Major Promotor of Breast Cancer Cell Aggressiveness
Source: Cancers (Basel). 2025 May 27;17(11):1793. doi: 10.3390/cancers17111793 (PMC12153661; doi:10.3390/cancers17111793)

Supplementary figures

Figure S1. Effect of lactate treatment on the expression of other markers in ER + breast cancer cells. Densitometric analysis of protein profiling for various targets in YS1.2 cells, either UT (solid bars) or in response to lactate treatment (open bars). Asterisks denote the significant differences compared to UT with * $p < 0.05$, ** $p < 0.001$ (n = 4 per group).

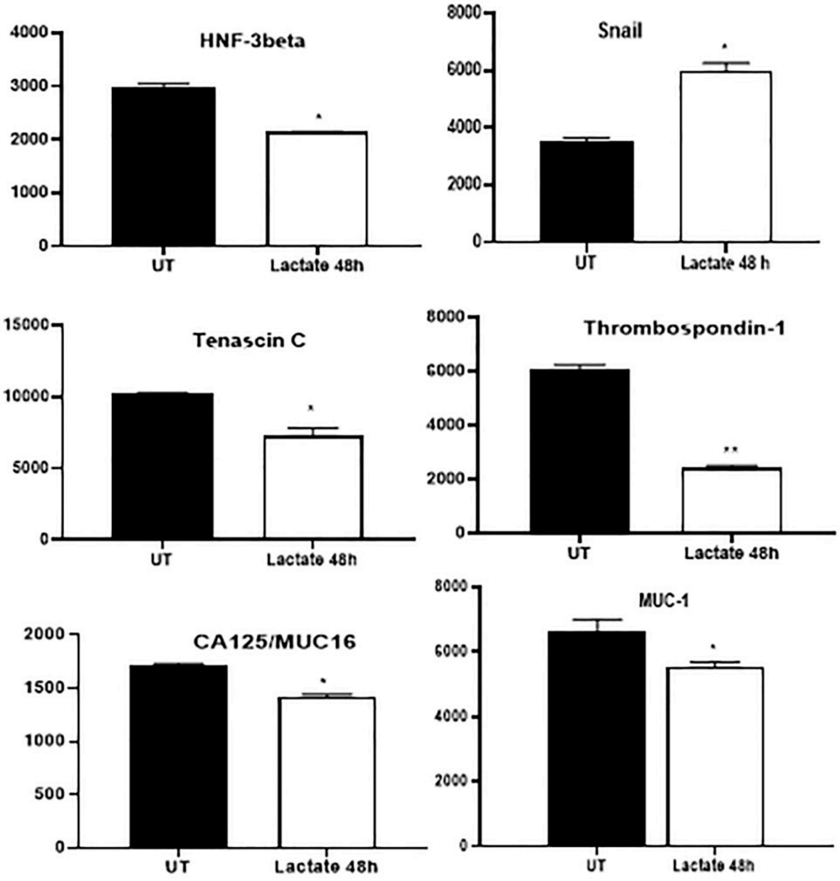

Figure S2: Effect of LDHB KO on the expression of other markers in pII cells. Densitometric analysis of protein profiling for various targets in pII cells, either UT (solid bars) or in response to LDHB KO (open bars). Asterisks denote the significant differences compared to UT with * $p < 0.05$, ** $p < 0.001$ (n = 4 per group).

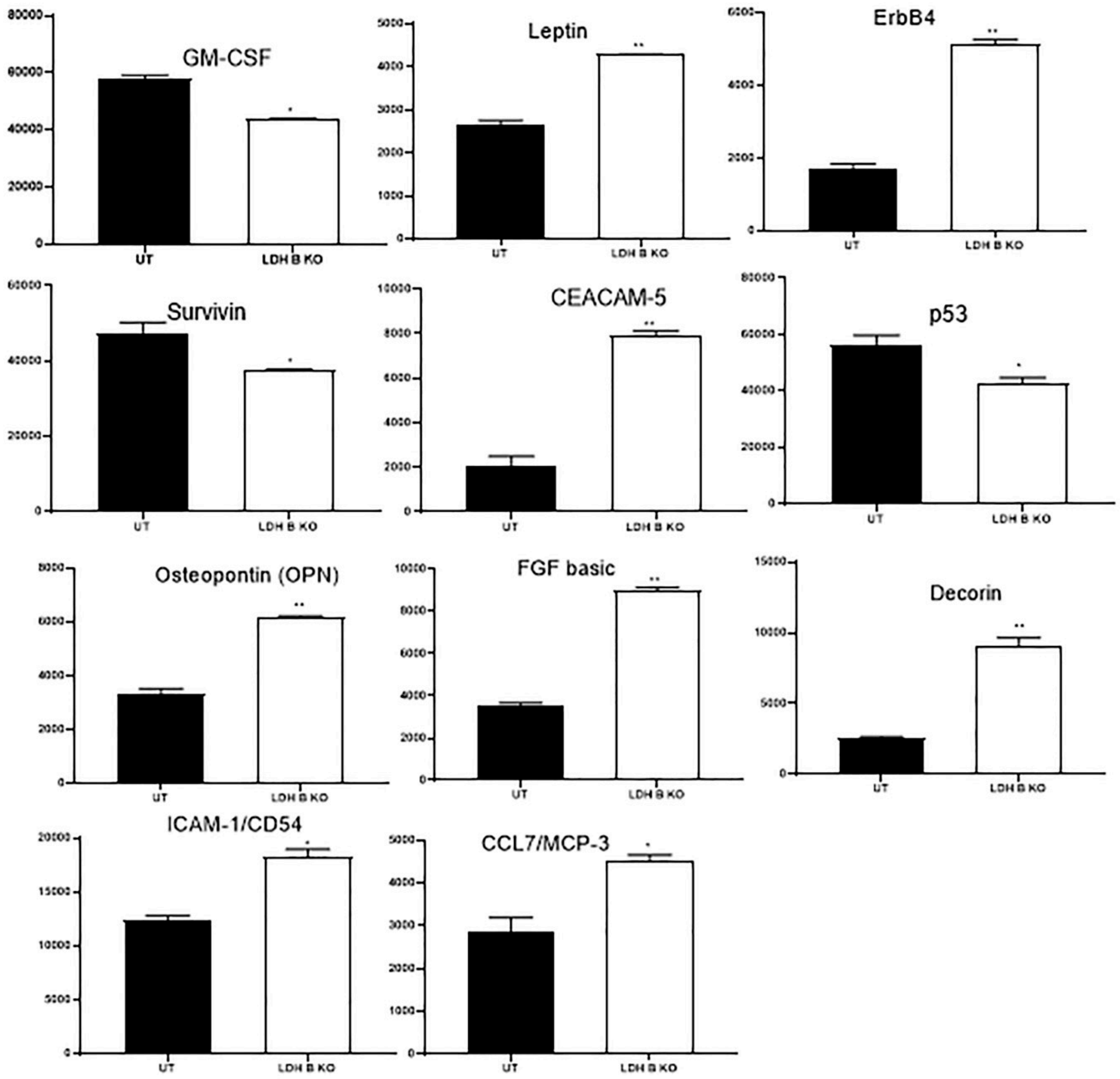

Figure S3: Effect of LDHB KO on the expression of other markers in MDA-MB-231 cells. Densitometric analysis of protein profiling for various targets in MDA-MB-231 cells, either UT (solid bars) or in response to LDHB KO (open bars). Asterisks denote the significant differences compared to UT with $*p < 0.05$, $**p < 0.001$, $***p < 0.0001$ (n = 4 per group).

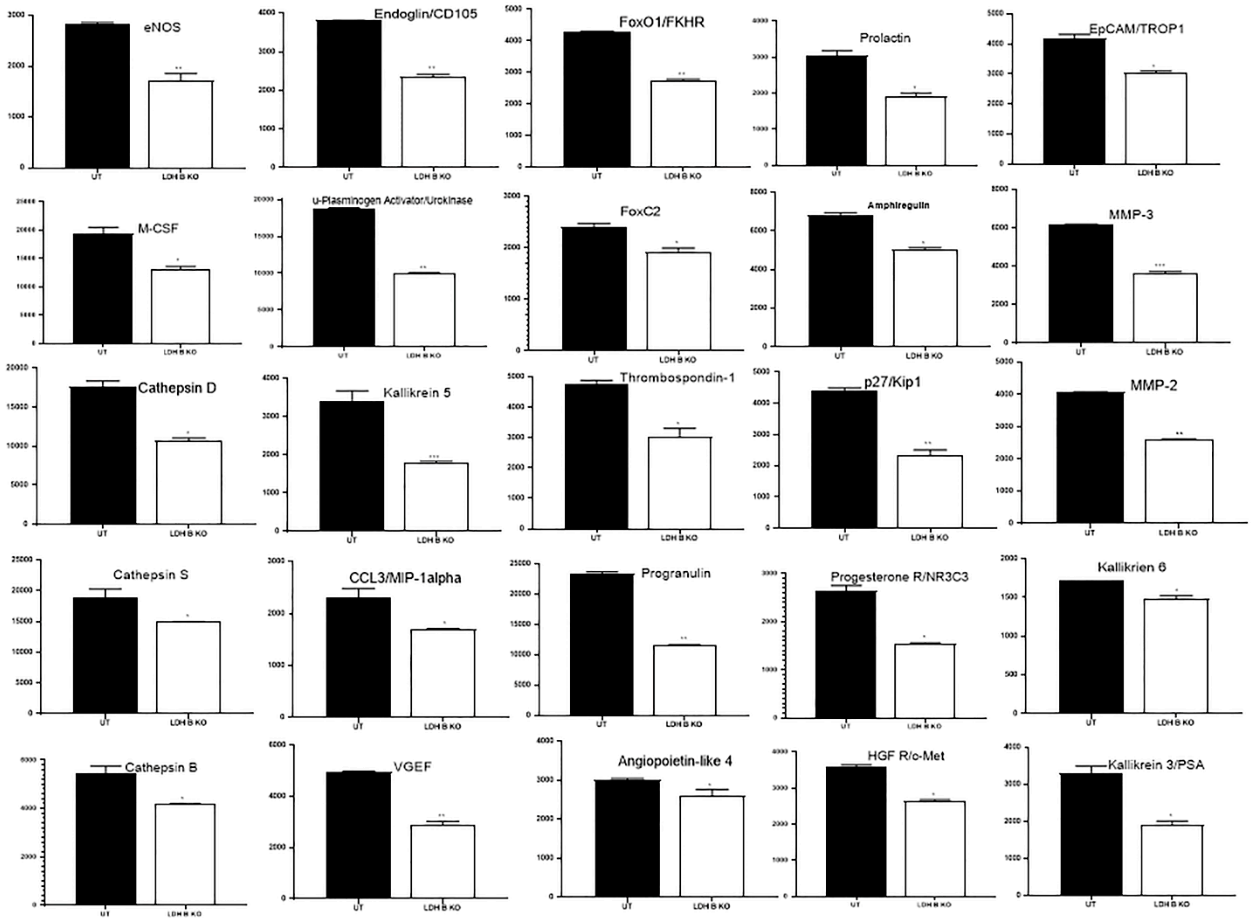

Supplement: Supplementary file 1 [file cancers-17-01793-s001.zip › cancers-3643604-Supplementary Materials.pdf]
