# Supplementary figures and images for: Lactate Is a Major Promotor of Breast Cancer Cell Aggressiveness
Source: Cancers (Basel). 2025 May 27;17(11):1793. doi: 10.3390/cancers17111793 (PMC12153661; doi:10.3390/cancers17111793)

Fig 2A

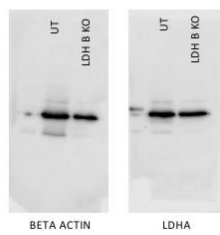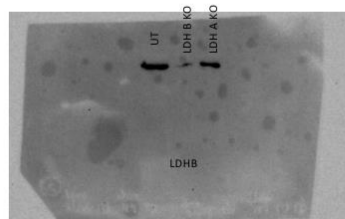

Fig 3D

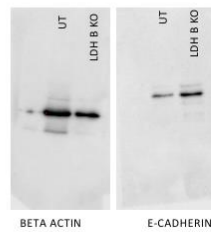

Fig 2A and 3D are from the same experiment that's why actin blot is the same

Fig 3C

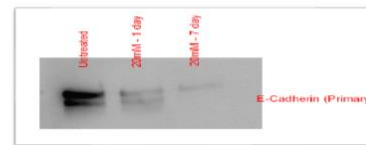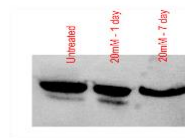

Actin

Supplement: Supplementary file 1 [file cancers-17-01793-s001.zip › cancers-3643604-File S1.pdf]
